# Problems in management of medication overuse headache in transgender and gender non-conforming populations

**DOI:** 10.3389/fneur.2024.1320791

**Published:** 2024-01-30

**Authors:** Cameron I. Martinez, Erika Liktor-Busa, Tally M. Largent-Milnes

**Affiliations:** Department of Pharmacology, College of Medicine, University of Arizona, Tucson, AZ, United States

**Keywords:** migraine, medication overuse headache, transgender, LGBTQ+, mental health, substance abuse disorder, chronic pain

## Abstract

Primary headache disorders, such as migraine, account for a significant portion of disability rates worldwide, yet patients still struggle to receive the adequate medical and emotional support necessary to improve health outcomes. Insufficient pain management through either impractical pharmaceutical treatments or absent emotional support networks can worsen physical and mental health outcomes since comorbidities commonly associated with headache include hypertension, diabetes, depression, and anxiety. A lack of awareness on headache pathology and its observable severity can lead to pain-related prejudice that destroys beneficial aspects of patient self-advocacy and self-efficacy, thus potentially discouraging the use of healthcare services in favor of maladaptive coping skills. Acute treatments for primary headache disorders include non-steroidal anti-inflammatory drugs (i.e., aspirin, ibuprofen), triptans (i.e., sumatriptan), and opioids; however, continuous use of these pain-relieving agents can generate a secondary headache known as medication overuse headache (MOH). Recent work highlighting the overlap of morphological and functional brain changes in MOH and substance use disorder (SUD) suggests that insufficient pain management encourages analgesic misuse. The LGBTQ+ community—specifically transgender and gender non-conforming persons—struggles with high rates of mental illness and substance abuse. Since gender-affirming sex hormone therapy influences migraine progression, transgender and gender non-conforming (trans*) patients on hormone therapy have a higher risk for worsening migraine symptoms. However, trans* patients are less likely to have access to appropriate pain management techniques, thus preventing positive health outcomes for this vulnerable population.

## 1 Introduction

In 2021, the Center for Disease Control reported that 51.6 million people (20.9%) in the United States experienced chronic pain, referring to any condition in which aberrant pain lasts for three or more months (1). The World Health Organization recognizes primary headaches, such as migraine, as the most disabling neurological disorder impacting an estimated 15% of the global population (2). Although the disparity in pain disorders implies multiple physiological mechanisms contribute to nociception, healthcare providers often address chronic pain as a singular symptom, suggesting a lack of treatment efficacy. Since patients tend to rely on prescription analgesics for long-term pain management, improper use could facilitate worsening comorbidities. Thus, research into the pronociceptive mechanisms of various pain disorders is needed to improve treatment efficacy.

Women are disproportionately burdened with chronic pain disorders, often reporting higher pain intensities and disability than men, but they still struggle to receive proper validation and treatment (3, 4). From this sex disparity, research has implicated estrogen hormones in propagating chronic pain disorders, including migraine in which pubertal hormones cause a sex-specific shift in incidence (5, 6). Transgender and gender non-conforming (trans*) individuals require gender-affirming healthcare utilizing sex hormone replacement therapy (HRT), but poor social acceptance hinders safe access to healthcare services. Therefore, this population suffers with significant unmet healthcare needs that have yet to be fully elucidated by research, but pre-existing mental health and evidence regarding sex HRT in trans* populations on migraine pathology can provide some insight (7, 8). To encourage further research on the physical healthcare needs of LGBTQ+ patients, this review examines analgesia-induced comorbidities in migraine and trans* populations to determine potential problems in pharmaceutical interventions and emotional support related to chronic pain management.

In headache disorders, the frequent consumption of analgesics can promote a comorbid secondary headache known as medication overuse headache (MOH) (9). The International Headache Society defines MOH as headache episodes occurring on 15 or more days when abortive medication is taken excessively and/or regularly for at least three months. MOH can manifest as a worsening primary headache or as a coinciding secondary headache (10). The probability of MOH increases with the rate/intensity of primary headache episodes; thus, patients with chronic migraine have the highest MOH incidence (11). Buse et al. (12) and Westergaard et al. (13) reported that approximately half of chronic migraineurs experienced MOH headaches. Furthermore, Lanteri-Minet et al. (14) noted that MOH in chronic migraineurs resulted in poorer quality of life and greater disability, thereby illustrating how inadequate pain management leads to worsening health outcomes (15). The features of MOH headache—particularly increased headache frequency, expansion of head/facial pain, and cutaneous allodynia—suggest that MOH utilizes the same molecular mechanisms underlying migraine to advance chronic pain (16).

Migraine relies on maladaptive neurovascular mechanisms such as central sensitization, endothelial dysfunction/oxidative stress, and reduced serotonin (5-HT) serum levels to enhance trigeminal nociceptive neurotransmission. Central sensitization is a key mechanism behind primary headache and is defined as abnormal cortical hyperexcitability within the central nervous system. Cortical networks are strengthened through recurring neuronal activation and synaptic plasticity induced by long-term potentiation to generate associations of headache pain with non-noxious environmental stimuli (17). Treatment guidelines for primary headache disorders outline different medications for preventative (reducing headache frequency) or abortive (eliminating headache pain and symptoms) pain relief. Abortive migraine medication includes triptans and opioids.

Triptans are generally recommended as the primary intervention for migraine and moderate-to-severe headache pain since they effectively reduce neurovascular dysfunction by temporarily inhibiting pain-promoting processes (18, 19). Triptans (i.e., sumatriptan) are serotonin receptor agonists that bind to 5-HT1B and 5-HT1D receptors and induce cerebral vasoconstriction by blocking the release of vasoactive neuropeptides, stabilizing neurovascular activity (20). However, persisting central sensitization can re-establish and even increase trigeminal pain through continuous synaptic plasticity, resulting in expanded neuronal receptive fields that amplify headache into MOH features (21). This triptan-induced latent sensitization can significantly diminish drug efficacy because cortical hyperexcitability continuously promotes nociception; therefore, frequent use can promote central sensitization as headache reoccurrence offsets periods of migraine relief. De Felice et al. (22) investigated triptan-induced latent sensitization and cutaneous allodynia in animal models. Calcitonin gene-related peptide and neuronal nitric oxide synthase—which are biomarkers associated with neurogenic inflammation and oxidative stress, respectively, in migraine—were upregulated in trigeminal ganglia dural afferents following triptan sensitization, supporting the theory that MOH enhances chronic pain through migraine processes (16).

Despite their potency, opioids are not recommended to treat chronic pain or primary headache disorders; however, 35% of migraine patients have received prescription opioids (23). Opioid use trends higher among chronic migraineurs and those with comorbid MOH, but rather than improving outcomes, opioids worsen headache-related disability (23). Caponnetto et al. (24) reported a two-fold risk for MOH following opioid use that increased to a three-fold risk in patients with a history of MOH and/or substance misuse. Opioids are analgesic compounds that bind to three types of G-protein coupled receptors (mu, delta, and kappa) to exert either agonist, antagonist, or partial agonist effects (25). While the specific mechanism of action can vary, activated G-protein coupled receptors typically trigger cellular hyperpolarization and reduce neurotransmitter release; therefore, opioid receptors within pain-processing cortical regions—such as the periaqueductal gray area (PAG), locus coeruleus, and rostral ventral medulla—could theoretically mitigate headache pain (25). Opioid-induced analgesia within the central nervous system is thought to occur from the activation of mu receptors in the midbrain, which indirectly stimulate descending inhibitory pathways within the PAG and nucleus raphe to reduce nociceptive neurotransmission through the dorsal horn (25). Much like triptans, opioid analgesia via inhibitory neurotransmission is insufficient to abolish migraine headache.

## 2 Medication overuse headache and mental health

Chronic pain often corresponds to comorbid mental health issues. Mood disorders such as anxiety and depression are two to ten times more common in migraine than in the general population (26). The severity of comorbid psychiatric disorders also tends to accurately predict features of migraine prognosis such as quality of life, headache frequency, and potential suicide risk (26).

Chronic pain disorders fall under the umbrella of invisible illnesses wherein symptoms are not inherently observable from an external perspective; therefore, a patient’s loved ones might find it difficult to fully conceptualize headache-related disability with its perceived inconsistencies, resulting in insufficient social support or prejudice by dismissing/downplaying pain severity (27). The incongruence between patient disability and perceived dysfunction from social support networks increases with headache frequency such that compared to other primary headaches, chronic migraineurs are less likely to rely on friends/family to provide adequate support and validation (28). Emotional and physical loneliness are also common as patients are often forced to take full responsibility over their health and its consequences (28); furthermore, the continued gaps in pain pathology research can foster distrust between patients and medical practitioners when healthcare professionals fail to properly address patient concerns (29). In the absence of real-life support, patients often turn to social media to build connections, understand their illness, and receive emotional validation. Although online relationships are generally a poor substitute for the possible benefits gained from physical relationships, online discussions about chronic illness with other patients may provide better support and resources that are then crucial to restore patient self-esteem, personal identity, and self-advocacy (28). Belot et al. (27) reported that migraineurs were more likely to have an insecure attachment style, fewer people included in their social networks, and less satisfaction obtained from social connections than the general population; therefore, successful chronic pain management requires coping skills to navigate the emotional trauma of chronic pain and reduce the risk of mental illness.

Due to the gap between disability and pain-related stigma, the absence of healthy coping skills beyond analgesics can encourage medication dependency that promotes MOH comorbidity. Compared to some well-known preventative health measures such as regular physical activity and nonsmoking status, successful social support operates both as a critical pain management technique and as a protective agent against further illness (30). Among migraineurs with comorbid MOH, poor social support positively correlated with disorders featuring more intense pain. Compared to the general population, chronic migraineurs had a higher rate of reduced social connection and loneliness while MOH migraineurs had the highest rate overall—especially concerning loneliness. Inadequate social support was characterized by infrequent contact with and fewer members belonging to a patient’s social network, but regardless of the amount of interaction, MOH migraineurs had persistent loneliness that likely reflects feelings of emotional isolation due to patient perceptions about their own ability to manage symptoms (30). Patients with chronic pain are fully capable of forming meaningful connections; however, the quality of these connections—particularly in their ability to provide compassionate support—is more essential towards managing patient wellbeing than the quantity. Westergaard et al. (30) proposed that loneliness associated with chronic pain limits the use of active coping skills for pain management while poor social support decreases patient self-efficacy. Therefore, in the same way that acquired MOH increases medication dependency as a direct result of enhanced pain, emotional grief associated with a lack of social connection can promote mental illness.

To mitigate the risk of MOH, proper use of analgesics is critical. However, because potent painkillers are often required to provide sufficient relief, the association between medication dependency in migraine and MOH can increase the risk of opioid addiction and/or substance abuse in some patients, especially those with a prior history (31). MOH seems to mimic substance use disorder (SUD) as both conditions demonstrate similar modifications in networks regulating motivation, reward, and behavioral control (16). When functional magnetic resonance imaging was used to compare analgesic medication to addictive drugs, noxious stimuli altered cortical activity in dopamine-related areas associated with pain and reward (21). Particularly, Li et al. (32) determined that MOH and SUD shared reduced gray matter in the insular lobe, dorsolateral prefrontal cortex, anterior cingulate cortex, and amygdala as well as increased functional connectivity in the salience network. Fuh et al. (33) and Radat et al. (34) examined MOH patients with chronic headache for substance abuse and determined that approximately 68%–70% met at least three out of five criteria for substance dependence as defined in the Diagnostic and Statistical Manual of Mental Disorders (DSM), fourth edition (35). Since the DSM-5 integrates substance dependence and abuse into severity symptoms for SUD, these patients would be diagnosed with mild SUD severity, suggesting that improper pain management facilitates inappropriate coping behaviors (36).

## 3 MOH comorbidities in LGBTQ+ and gender non-conforming populations

Given the overlap between chronic pain and SUD, the LGBTQ+ community is suspected to be at particular risk for poor pain outcomes since this population carries significant mental (i.e., depression, anxiety, SUD) and physical (i.e., chronic pain, neurological disorders, mobility impairment) health disparities (37, 38). Additionally, trans* patients face significant barriers in accessing necessary healthcare due to the potential risk for harassment, discrimination, or refusal of care (39). Increased difficulty in navigating the healthcare system can erode trust between LGBTQ+ patients and medical professionals, thereby encouraging the use of maladaptive coping skills for pain relief (39). To effectively treat the unmet healthcare needs of pain management, pharmacological treatments must be comprehensively conceptualized with the current understanding of pre-existing health disparities and healthcare barriers faced by queer patients.

Minority stress theory reliably predicts health disparities among marginalized groups since chronic stress from social stigma, discrimination, and violence promote deteriorating health outcomes (39). Given that migraineurs and trans* patients both experience poor social support from the public and healthcare system, these populations require comprehensive pain management that acknowledges the multidimensional aspects of physical and emotional pain. Although the exact incidence of primary headache remains unknown, trans* individuals might be particularly susceptible to migraine due to gender-affirming sex hormones and their implications on migraine pathology [refer to Martinez et al. (7)]. Furthermore, analgesic efficacy can vary by gender as investigated through biological sex differences; while sex differences in pain perception cannot be concretely determined, pain disorders are more common among women due to estrogen-induced nociception. Aloisi et al. (40) examined pain perception following sex hormone HRT among trans* men and women and determined that higher estrogen levels corresponded to novel/worsening pain with increased sensitivity correlating to HRT duration.

The ability for patients to achieve pain relief depends on their mental health; therefore, the burden of mental illness among LGBTQ+ patients combined with significant marginalization perpetuates negative pain outcomes. In 2013 the Substance Abuse and Mental Health Services Administration (SAMHSA) reported a 6.7% prevalence of clinical depression in the general population (41). Among a population of transgender men and women, Bockting et al. (42) found that 44.1% had clinical depression while 33.2% had an anxiety disorder. For gender non-conforming trans* individuals, Gordon et al. (38) determined that 14% had moderate-to-severe levels of depression and anxiety with gender non-conformity demonstrating a modest positive correlation on mental health severity. In 2010, the National Center for Transgender Equality and the National Gay and Lesbian Task Force found that 8% of trans* participants were actively misusing drugs and alcohol to cope with gender-related stigma while an additional 18% had a history of substance abuse (43). Compared to the NIH’s estimate in 2009, 7.3% of the general population abused/depended on alcohol while 1.7% abused non-prescription drugs. When LGBTQ+ patients experience discrimination from healthcare services, they are more likely to rely on prescription medication or addictive substances. In 2018, SAMHSA reported an 8% prevalence of analgesic and opioid misuse among gender non-conforming persons, which is double the rate among the general population (43). Restar et al. (44) highlighted the consequences of transmisogyny (intersection of transphobia and misogyny experienced by transfeminine people) since trans* women had a lifetime prevalence of 11.8% for substance misuse that compares to 12.6% among cisgender (gender identity aligns with assigned sex) populations (45). Furthermore, Kidd et al. (46) reported that chronic exposure to prejudice and discrimination increases the risk of prescription medication abuse, further supporting the notion that medication management is influenced by physical and emotional distress (45).

## 4 Discussion

**Figure 1:**
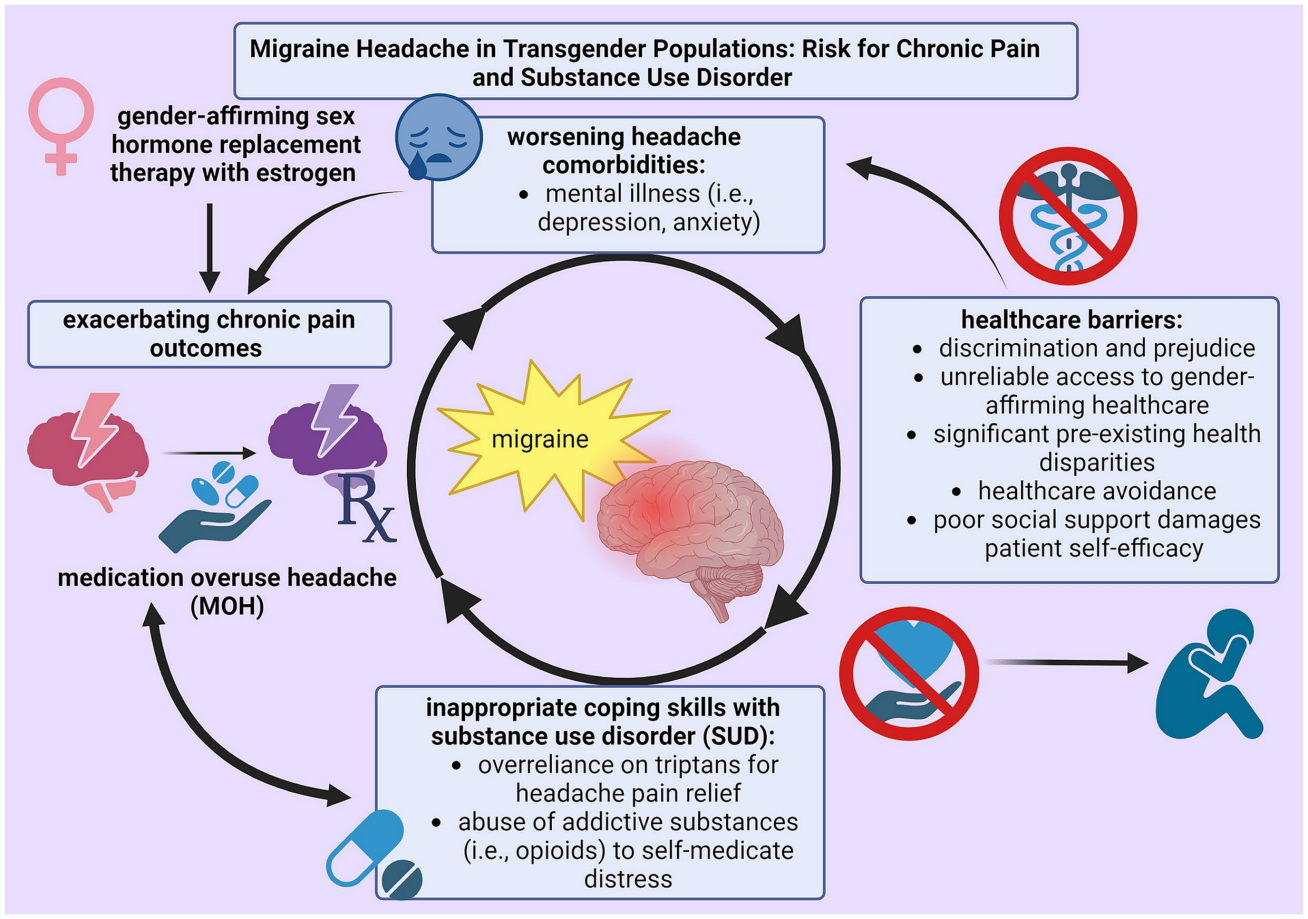
Cycle of self-perpetuating negative physical (chronic pain) and mental (substance use disorder) health outcomes as a result from insufficient techniques for pain management among trans* patients with migraine.

Trans* patients are proposed to have a high incidence of primary headache disorders because outstanding health disparities, lack of supportive health measures, inability to access healthcare services, and encountered prejudice uphold detrimental health outcomes. Pre-existing health disparities among trans* patients not only hinder successful pain management by eliminating protective health measures, but also expediate disability by advancing pain severity (38, 47). The substantial impact of SUD within LGBTQ+ populations is especially damaging because assumptions about migraine medication efficacy can encourage frequent use and potential abuse. Because analgesics are associated with pain relief and the burden of disease within LGBTQ+ migraineurs is extensive due to multiple biopsychosocial factors, SUD actively cultivates inappropriate techniques to handle chronic illness. Figure 1 illustrates this phenomenon. Therefore, research into the prevalence and treatment of MOH in this migraine subpopulation is critical to improve health outcomes.

Healthcare attitudes regarding the treatment of trans* patients remain a crucial element in promoting positive health outcomes, and current efforts to improve LGBTQ+ relations with medical professionals have mostly focused on LGBTQ+-centered education to minimize prejudice and discrimination (48). However, this method only addresses the primary barrier that prevents trans* patients from accessing healthcare services. Tordoff et al. (49) demonstrated that gender-affirming healthcare significantly improves rates of depression and suicidal thoughts by 60% and 70%, respectively; thus, the lack of research examining the influence of sex HRT on pain reinforces inferior outcomes by disregarding patient concerns about the influence of gender identity on physical health (39). Although gender-affirming healthcare is considered medically necessary for this population, legislative efforts to eliminate access to gender-related services have risen within recent years. The American Civil Liberties Union reported that as of February 7th, 2023, 88 bills attacking gender-affirming healthcare have been introduced across 26 states with many attacking trans* youth and healthcare professionals involved in gender-affirming services (50). These legal and social barriers within healthcare settings are crucial issues that must be addressed to facilitate long-term positive health outcomes. Therefore, as an ongoing effort to improve the understanding of LGBTQ+ healthcare needs, trans* patients must have access to gender-affirming medical treatment despite the possible risk for acquired chronic pain, substance abuse disorder, and mental illness.

## 5 Conclusion

While the reliance on analgesics for chronic pain can worsen health outcomes, healthcare professionals should understand and be able to explain the efficacy and shortcomings of different pharmaceutical interventions to prevent MOH and SUD. Further research on chronic pain and primary headache pathology is needed to improve analgesics that target specific pronociceptive mechanisms. Future studies investigating the incidence of chronic pain and headache within trans* populations are critical to determine the current severity of unmet healthcare needs present in this population; additionally, particular attention should be directed to sex hormones and their involvement in pronociceptive mechanisms to determine and prevent potential risks from gender-affirming HRT. Improved pharmaceuticals can then be tailored towards mitigating the response between hormones, patient needs, and nociception. Overlaps between migraine and trans* patients demonstrate that access to healthcare services is not enough to encourage positive pain outcomes, especially when physical symptoms are compounded with emotional distress. To mitigate barriers contributing to healthcare avoidance and to prevent the use of addictive substances for pain relief, chronic pain management requires subsequent mental health support centered on emotional validation, patient self-efficacy, and personal identity.

## Author contributions

CM: Conceptualization, Data curation, Formal analysis, Visualization, Writing – original draft, Writing – review & editing. EL-B: Validation, Writing – review & editing. TL-M: Conceptualization, Project administration, Supervision, Validation, Visualization, Writing – review & editing.
